# Supplementary figures and images for: Toxoplasma gondii Co-opts the Unfolded Protein Response To Enhance Migration and Dissemination of Infected Host Cells
Source: mBio. 2020 Jul 7;11(4):e00915-20. doi: 10.1128/mBio.00915-20 (PMC7343987; doi:10.1128/mBio.00915-20)

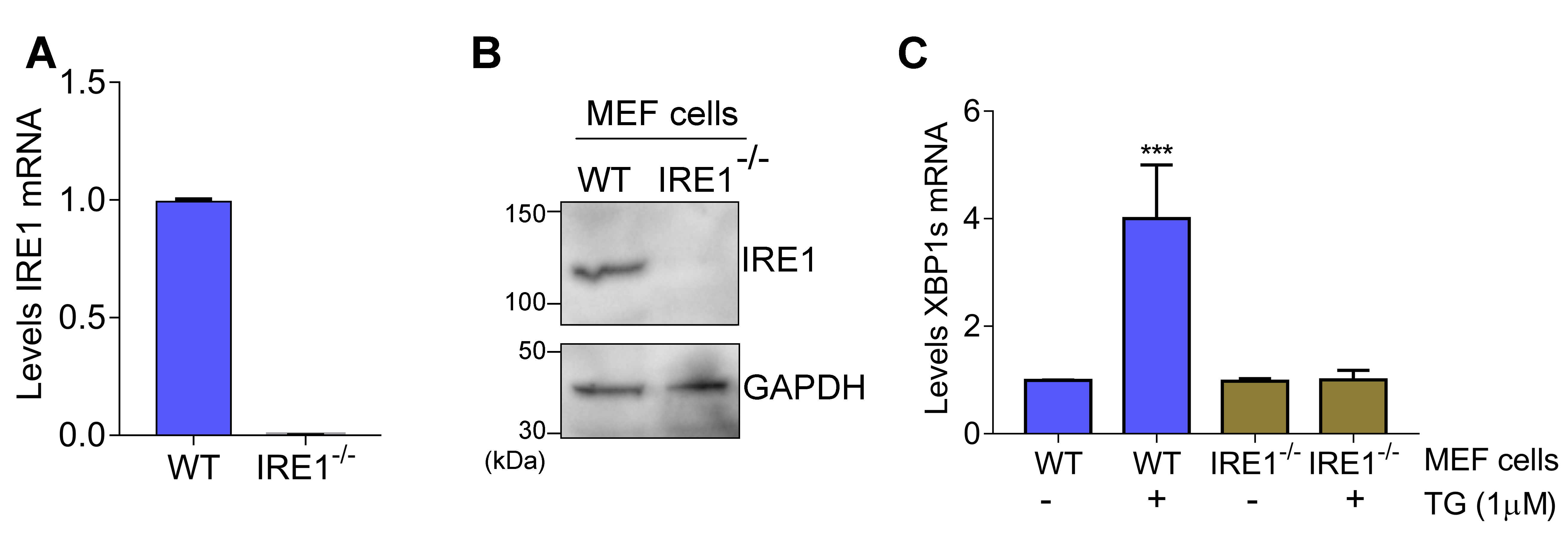

Supplement: FIG S1 [file mBio.00915-20-sf001.tif]

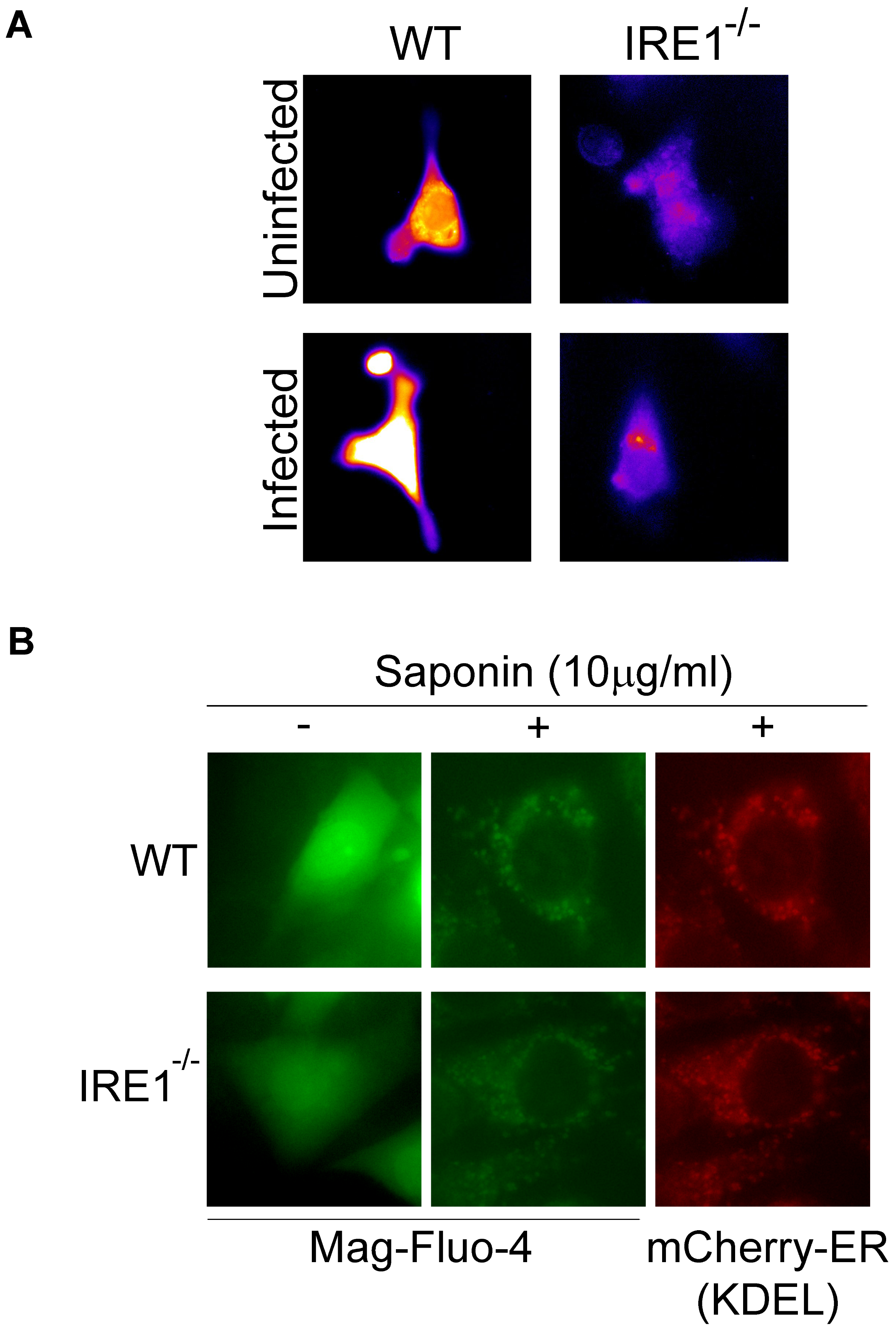

Supplement: FIG S2 [file mBio.00915-20-sf002.tif]

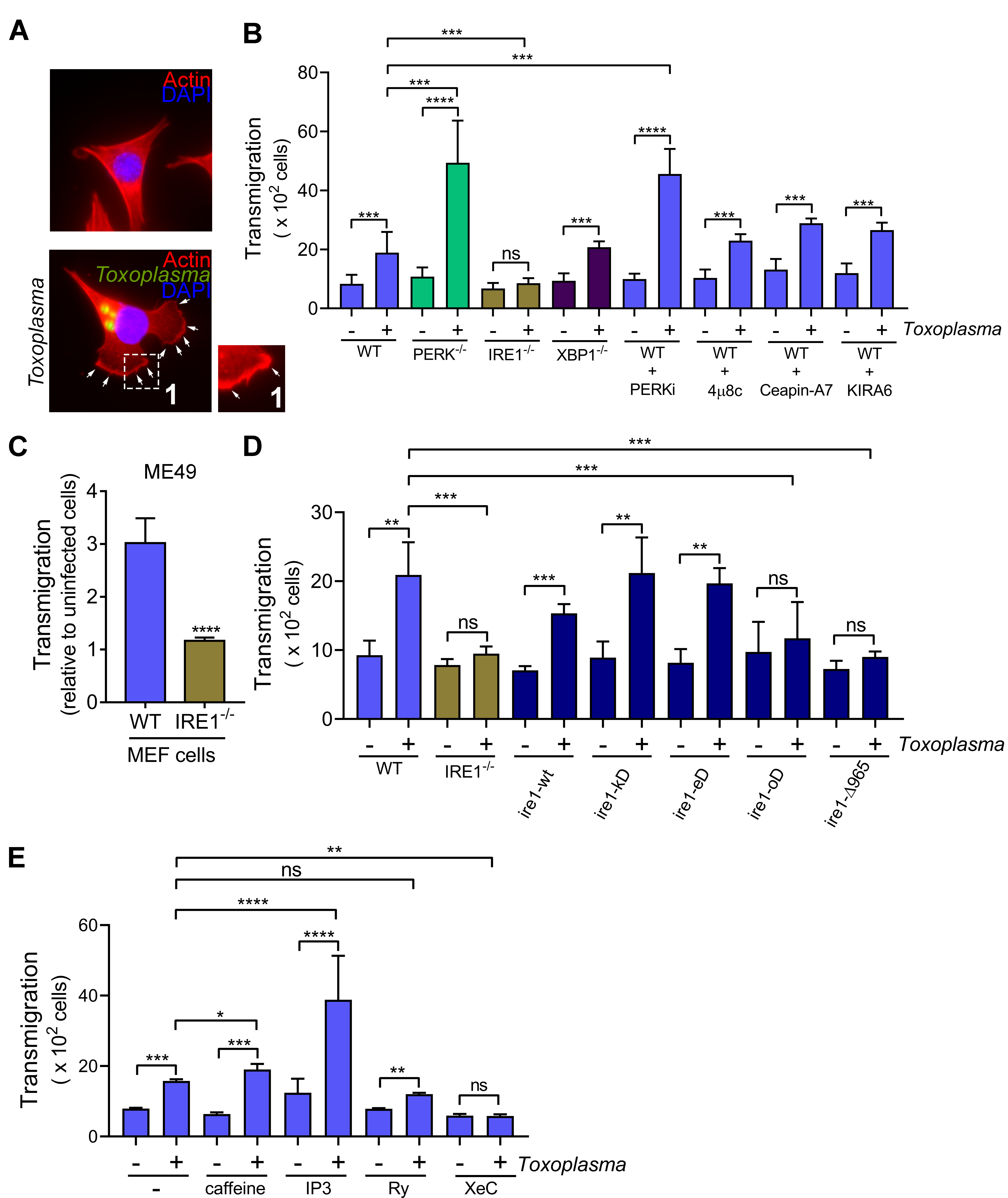

Supplement: FIG S3 [file mBio.00915-20-sf003.tif]

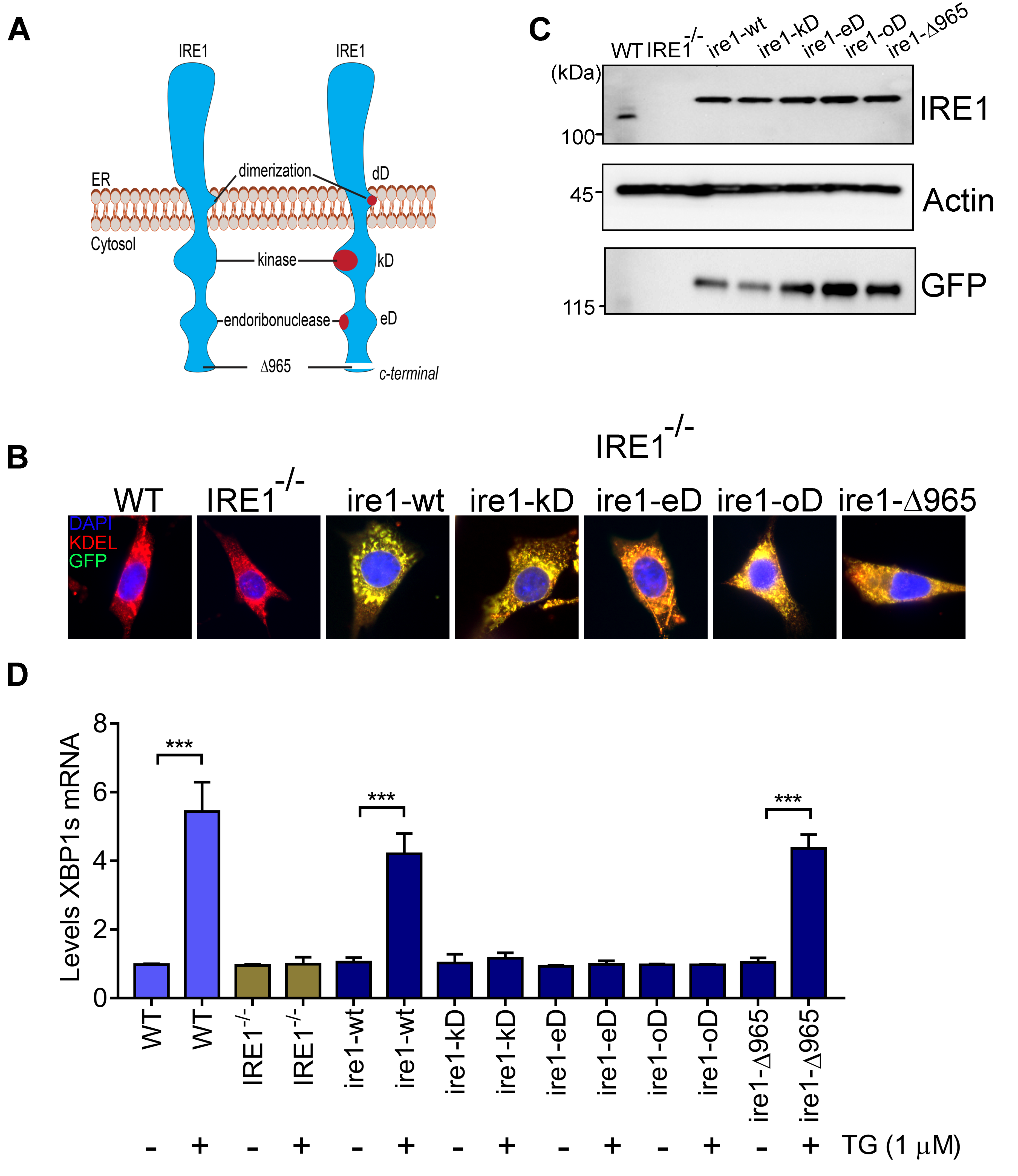

Supplement: FIG S4 [file mBio.00915-20-sf004.tif]

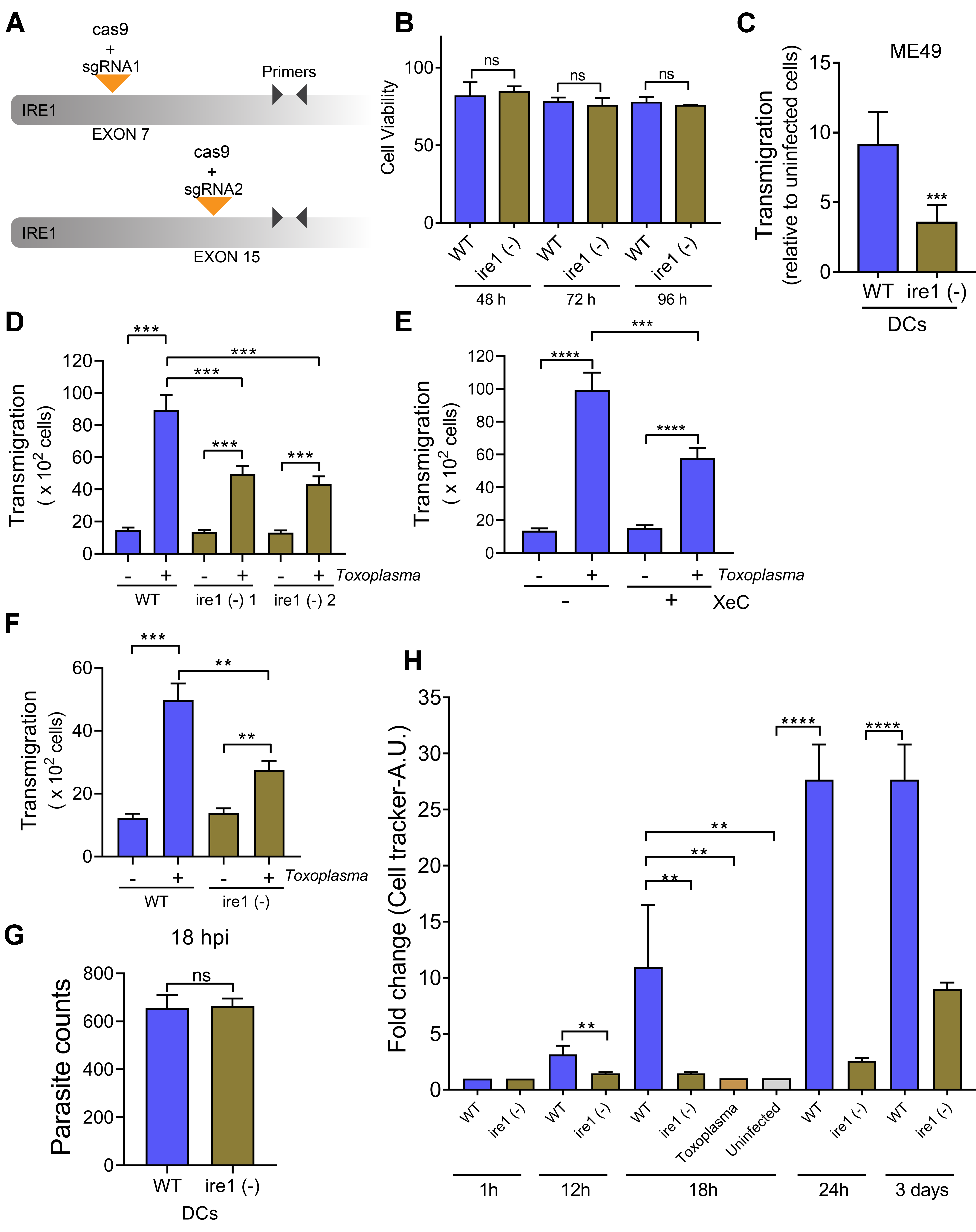

Supplement: FIG S5 [file mBio.00915-20-sf005.tif]
