# Supplementary material for: Toxoplasma gondii Co-opts the Unfolded Protein Response To Enhance Migration and Dissemination of Infected Host Cells
Source: mBio. 2020 Jul 7;11(4):e00915-20. doi: 10.1128/mBio.00915-20 (PMC7343987; doi:10.1128/mBio.00915-20)
Supplement: TABLE S1 [file mBio.00915-20-st001.docx]

**Supplementary Table 1. Oligonucleotide primers used in this study.**

| Primer | 5’—3’ |
| --- | --- |
| *Xbp1-F* | ACATCTTCCCATGGACTCTG |
| *Xbp1-R* | TAGGTCCTTCTGGGTAGACC |
| *Xbp1u-F* | GAAGAGAACCACAAACTCCAGC |
| *Xbp1u-R* | GCAGAGGTGCACATAGTCTGAG |
| *Xbp1s-F* | GAGTCCGCAGCAGGTG |
| *Xbp1s-R* | TCCAGAATGCCCAAAAGG |
| *Edem-F* | GGGACCAAGAGGAAAAGTTTG |
| *Edem-R* | GAGGTGAGCAGGTCAAATCAA |
| *Hrd1-F* | AGCTACTTCAGTGAACCCCACT |
| *Hrd1-R* | CTCCTCTACAATGCCCACTGAC |
| *Grp78-F* | TGTGGTACCCACCAAGAAGTC |
| *Grp78-R* | TTCAGCTGTCACTCGGAGAAT |
| *Erdj4-F* | CTTAGGTGTGCCAAAGTCTGC |
| *Erdj4-R* | GGCATCCGAGAGTGTTTCATA |
| *P58ipk-F* | GTGGCATCCAGATAATTTCCAG |
| *P58ipk-R* | GAGTTCCAACTTCTGTGGAAGG |
| *Pdia6-F* | TGGTTCCTTTCCTACCATCACT |
| *Pdia6-R* | ACTTTCACTGCTGGAAAACTGC |
| *Ero1l-F* | CGGACCAAGTTATGAGTTCCA |
| *Ero1l-R* | TCAGAGAGATTCTGCCCTTCA |
| *Ire1-wt-F* | TCTAGAACCATGCCGGCCCGGCGGCTGCTGCTGCTGCTGAC |
| *Ire1-wt-R* | GAGGGCGTCTGGAGTCACTGGGGGCTGGGGCTCTGGGGGCTCG |
| *Ire1-kD-F* | GCGACGTGGCCGTGAGGATCCTCCCCGAG |
| *Ire1-kD-R* | GGTTGTCAAACATGCCCCGGTACA |
| *Ire1-eD-F* | CTCCGAGCCATGAGAGAAGCACCACTACCGGGAGCTGCC |
| *Ire1-eD-R* | GAGATCTCTGACAGAACCACCTTTAT |
| *Ire1-oD-F* | GGTAAAAAGCAGATCTGGTATGTTATTGACCT |
| *Ire1-oD-R* | CATGTAGAGGATTCCATCTGAACTTCGGCATG |
| *Ire1-* *Δ965-F* | TGAGCGAGGGCGGCCCC |
| *Ire1-* *Δ965-R* | CAGCTCCCGGTAGTGGTGCTTCTTATTTC |
| *Gapdh-F* | TCACCACCATGGAGAAGGC |
| *Gapdh-R* | GCTAAGCAGTTGGTGGTGCA |
