# Supplementary material for: Toxoplasma gondii Co-opts the Unfolded Protein Response To Enhance Migration and Dissemination of Infected Host Cells
Source: mBio. 2020 Jul 7;11(4):e00915-20. doi: 10.1128/mBio.00915-20 (PMC7343987; doi:10.1128/mBio.00915-20)
Supplement: TABLE S2 [file mBio.00915-20-st002.docx]

**Supplementary Table 2. Reagents used in this study.**

| Reagent | Company names |
| --- | --- |
| **Antibody** | |
| PERK-P | Phospho-PERK (Thr980) (16F8) Rabbit mAb #3179- Cell Signaling |
| PERK | PERK (C33E10) Rabbit mAb #3192- Cell Signaling |
| ATF6 | [30] |
| XBP1s | XBP-1s (D2C1F) Rabbit mAb #12782- Cell Signaling |
| GAPDH | ab9485-Abcam |
| Phalloidin | R415-Thermo Fisher Scientific |
| SAG1 | Toxoplasma gondii P30 Monoclonal Antibody (P30/3)- Thermo Fisher Scientific |
| IRE1 | ab37073-Abcam |
| Myc | Myc-Tag (71D10) Rabbit mAb #2278-Cell Signaling |
| GFP | clone GFP-20-Sigma Aldrich |
| Filamin A-P (S2152) | Phospho-Filamin A (Ser2152) Antibody #4761-Cell Signaling |
| Filamin A | Filamin A Antibody #4762-Cell Signaling |
| **Reagent** | |
| Fluo-4, AM | F14201-Thermo Fisher Scientific |
| Ry | 1329-Tocris |
| XeC | (-)-Xestospongin C- 1280-Tocris |
| IP_3_ | D-myo-Inositol 1,4,5-trisphosphate- 1482-Tocris |
| caffeine | C53-Sigma Sigma Aldrich |
| Mag-Fluo-4 | M14206- Thermo Fisher Scientific |
| PERKi | GSK2656157- 5.04651-Sigma Aldrich |
| 4µ8c | 4479-Tocris |
| Ceapin-A7 | SML2330-Sigma Aldrich |
| KIRA6 | 19151-Cayman Chemical |
| Collagen I | A1048301-Gibco |
| Thapsigargin | 1138-Tocris |
| DAPI | D9542-Sigma Aldrich |
